# Genetic alterations of *TP53* and *OTX2* indicate increased risk of relapse in WNT medulloblastomas: “it’s a numbers game”—implications for WNT medulloblastoma dose-reduction clinical trials

**DOI:** 10.1007/s00401-022-02518-0

**Published:** 2022-11-20

**Authors:** Nicholas G. Gottardo

**Affiliations:** 1 Department of Pediatric and Adolescent Oncology and Hematology, Perth Children’s Hospital, Perth, Western Australia Australia; 2 Brain Tumor Research Program, Telethon Kid’s Cancer Centre, Telethon Kid’s Institute, University of Western Australia, Perth, Western Australia Australia

Goschzik and colleagues [3] identified *TP53* mutation and *OTX2* gain as independent prognostic markers in WNT medulloblastoma patients. Findings were derived from 191 (of which 120 were clinically annotated) pediatric and adult retrospective WNT medulloblastoma cases in the German HIT-2000 trial and HIT registries. Based on these findings, the authors propose *TP53* mutation and *OTX2* gain as biomarkers to identify WNT patients at higher risk of relapse, which in their series comprised 58.1% of patients. The authors should be commended on gathering the largest WNT medulloblastoma cohort to date; however, the retrospective nature and heterogeneous treatments limit the interpretation.

These findings are important because no molecular biomarkers to refine stratification for WNT medulloblastoma exist. Overall survival (OS) of > 90% [1, 2, 4, 5] (Table 1) for WNT medulloblastoma has led to dose-reduction trials to try to limit long-term toxicity (Children’s Oncology Group (COG) ACNS1422 NCT02724579; SJMB12 NCT01878617; SIOP-PNET5 NCT02066220). Given relapsed WNT medulloblastoma is essentially incurable [6, 8], excluding patients with *TP53* mutation and/or *OTX2* gain would have important clinical implications.

**Table 1:** Survival and relapse pattern of WNT medulloblastoma patients by different cooperative groups and case series

Treatment approach	5-year event free survival	5-year overall survival	Relapse type *n* (%)			
			Local	Distant	Combined	Total
St Jude SJMB03 Phase III (*n* = 46) [2]	^&^100%	100%	0	0	0	0
COG ACNS0331 Phase III (*n* = 64) [5]	93.3%	NR	4 (100%)	0	0	4 4/64 (6%)
COG ACNS0332 Phase III (*n* = 14) [4]	92.9%	100%	NR	NR	1 (100%)	1 1/14 (7%)
HIT-SIOP-PNET4 Phase III (*n* = 58) [1, 8]	91%	NR	2 (25%)	4 (50%)	2 (25%)	8 8/58 (14%)
MAGIC & Int.CC cohorts (retrospective) (*n* = 101) [9]	NR	90%* vs 97%^#^ DC 86%* vs 94%^#^ VC	NR	NR	NR	NR
MAGIC & Int.CC cohorts (retrospective) (*n* = 93) [6]	^&^84%	92.9%	3 (20%)	12 (80%)	0	15 15/93 (16%)
Present series (retrospective) (*n* = 120) [3]	^&^68%* vs 93%^#^ ^&^72%^@^ vs 93%^^^	88%* vs 94%^#^ 82%^@^ vs 97%^^^	3 (27%)	6 (55%)	2 (18%)	11 11/120 (9%)

*DC* discovery cohort (*n* = 66), *VC* validation cohort (*n* = 35), *NR* not reported, *MAGIC* Medulloblastoma Advanced Genomics International Consortium, *Int.CC* International Collaborating Centers

^&^Progression free survival

*TP53 mutated

^#^*TP53* wildtype

^@^*OTX2* gain

^^^No *OTX2* gain

Previously, Zhukova et al. reported no significant difference in OS for *TP53* mutated versus wildtype WNT patients [9]. A recent study however reinvigorated interest in the prognostic relevance of *TP53* mutations in WNT medulloblastoma, reporting that four of five (80%) relapsed patients had *TP53* mutations [7]. Goschzik and colleagues identified *TP53* mutations in 16.1% (30/186) of WNT samples and support these findings, with six of eleven (54.5%) patients who relapsed harboring a *TP53* mutation (Table 2) [3]. The novel finding of *OTX2* oncogene gain in 42/108 (39%) WNT medulloblastomas was identified using molecular inversion probe (MIP) array technology and was associated with significantly worse progression free survival (PFS) and OS [3]. Overall, all 11 patients who relapsed harbored either a *TP53* mutation (3/11 (27%)) or *OTX2* gain [(5/11 (45%)) or both (3/11 (27%)] (Table 2). A sub-analysis of an “average-risk pure” cohort to mimic current dose-reduction clinical trial inclusion criteria resulted in the loss of statistical significance for survival outcomes of *TP53*-mutated patients. However, *OTX2* gain remained a significant indicator of OS and PFS, reinforcing the potential validity of this putative novel prognostic biomarker. Approximately 10% of medulloblastoma patients are WNT, with up to 18% (average 11%) of these harboring *TP53* mutations (Table 2). Such small patient numbers in *TP53*-mutated WNT cohorts suggest that both positive and negative results should be interpreted with caution. The statistical significance of findings represents the measure of confidence in the results, how likely or unlikely those results would have been observed by chance rather than because of a causative relationship; the *p* value per se neither confirms nor negates the data itself.

**Table 2:** Characteristics of WNT medulloblastoma patients by cooperative group clinical trial or case series

Characteristic	SJMB03 [2] (n = 53)	ACNS-0331 [5] (*n* = 64)	ACNS-0332 [4] (*n* = 14)	HIT-SIOP-PNET4 [1, 8] (*n* = 58)	MAGIC & Int.CC [9] (*n* = 101)	MAGIC and Int.CC [6] (*n* = 93)	UKCCSG, Int.CC and ICGC [7] (*n* = 29)	Present study [3] (*n* = 191)
No. of *TP53* mutations (%)	4/49 (8%)	1/37 (3%)	1/9 (11%)	NR	18/101 (18%)	9/86* (10%)	3/24 (13%)^$^ 4/5 (80%)^@^	30/186 (16%)
No. of *OTX2* amp/gain (%)	0	0	0	NA	NA	NR	NR	42/108 (39%)
Molecular technique used	DNAm, WES, GEP	DNAm, WES	DNAm, WES	iFISH, *CTNNB1* SS	*nanoString, TP53* SS	DNAm, GEP	DNAm, WES, TPS	DNAm, TPS, SS, MIP
Total no. of relapses	0	4	1	8	NR	15	5	11
No. of relapses with *TP53 *mutation	0	0	0	NR	NR	3/15 (20%)	4/5 (80%)	6/11 (54.5%)
No. of relapses with *OTX2*amp/gain^	0	0	0	NA	NA	NA	NA/NR	8/11^ (873%)

*NR* not reported, *NA* not assessed, *Amp* amplification, *UKCCSG* United Kingdom Children's Cancer Study Group, *MAGIC* Medulloblastoma Advanced Genomics International Consortium, *Int.CC* International Collaborating Centers, *ICGC* International Cancer Genome Consortium, *iFISH* interphase fluorescence in situ hybridization, *SS* Sanger sequencing, *DNAm* DNA methylation profiling, *GEP* gene expression profiling, *WES* whole exome sequencing, *MIP* molecular inversion probe arrays, *TPS* targeted panel sequencing

*17p loss (*TP53* mutation surrogate)

^$^Diagnostic WNT cohort

^@^Relapsed WNT cohort

However, some results of the current study appear inconsistent with recent prospective clinical trials [2, 4, 5]. In SJMB03 (where 8% of WNT patients were *TP53* mutated), 5-year PFS and OS were 100% [2]. In COG ACNS0331, only 3% (1/37) of the 64 WNT patients had a *TP53* mutation and 5-year EFS was 93.3%, with only 4 local failures [5], while in ACNS0332, 11% (1/9) had a *TP53* mutation and only one patient relapsed (Tables 1 and 2) [4]. Notably, no *TP53* mutations were identified in three of the four relapsed patients on ACNS0331 tested, nor the one relapsed patient from ACNS0332 (data provided by COG). One potential reason for this discrepancy may be due to the different therapies employed, negating *TP53* as a prognostic biomarker, as previously hypothesized [6]. None of the three studies [2, 4, 5 and data provided by COG] identified *OTX2* amplification/gain in WNT patients; however, MIP array technology was not utilized for any of these. Since copy number variations can be extracted from whole exome sequencing and DNA methylation profiling, the retrospective series which utilized these techniques could be re-examined [6, 7]. Moreover, for independent validation of the *OTX2* gain findings [3], additional analysis using MIP array could be considered in the SJMB03, ACNS0331 and ACNS0332 clinical trials cohorts. If confirmed, the feasibility of incorporating MIP into clinical practice must be evaluated.

In summary, combined with Richardson and colleagues’ report [7], these hypothesis-generating findings should be pursued further. Crucially, it will be important for the current front-line WNT dose-reduction trials to assess the two proposed biomarkers in diagnostic samples (and matched relapse samples, where available) from enrolled patients. A pooled analysis from these trials may also be required, which could additionally identify more biomarkers into the future.

## Data Availability

All presented data are published and publicly available or linked to the publications.
